# Clinical Benefits, Costs, and Cost-Effectiveness of Neonatal Intensive Care in Mexico

**DOI:** 10.1371/journal.pmed.1000379

**Published:** 2010-12-14

**Authors:** Jochen Profit, Diana Lee, John A. Zupancic, LuAnn Papile, Cristina Gutierrez, Sue J. Goldie, Eduardo Gonzalez-Pier, Joshua A. Salomon

**Affiliations:** 1 Baylor College of Medicine, Department of Pediatrics, Texas Children's Hospital, Section of Neonatology, Houston, Texas, United States of America; 2 Baylor College of Medicine, Department of Medicine, Section of Health Services Research, Houston, Texas, United States of America; 3 Harvard University, Harvard Initiative for Global Health, Cambridge, Massachusetts, United States of America; 4 Beth Israel Deaconess Medical Center, Department of Neonatology, Boston, Massachusetts, United States of America; 5 Harvard Medical School, Department of Pediatrics, Division of Newborn Medicine, Boston, Massachusetts, United States of America; 6 Mexican Ministry of Health, Economic Analysis Unit, México D.F., Mexico; 7 Harvard School of Public Health, Department of Health Policy and Management, Boston, Massachusetts, United States of America; 8 Harvard School of Public Health, Center for Health Decision Science, Boston, Massachusetts, United States of America; 9 Fundación Mexicana para la Salud, México D.F., Mexico; 10 Harvard School of Public Health, Department of Global Health and Population, Boston, Massachusetts, United States of America; University of Queensland, Australia

## Abstract

**Background:**

Neonatal intensive care improves survival, but is associated with high costs and disability amongst survivors. Recent health reform in Mexico launched a new subsidized insurance program, necessitating informed choices on the different interventions that might be covered by the program, including neonatal intensive care. The purpose of this study was to estimate the clinical outcomes, costs, and cost-effectiveness of neonatal intensive care in Mexico.

**Methods and Findings:**

A cost-effectiveness analysis was conducted using a decision analytic model of health and economic outcomes following preterm birth. Model parameters governing health outcomes were estimated from Mexican vital registration and hospital discharge databases, supplemented with meta-analyses and systematic reviews from the published literature. Costs were estimated on the basis of data provided by the Ministry of Health in Mexico and World Health Organization price lists, supplemented with published studies from other countries as needed. The model estimated changes in clinical outcomes, life expectancy, disability-free life expectancy, lifetime costs, disability-adjusted life years (DALYs), and incremental cost-effectiveness ratios (ICERs) for neonatal intensive care compared to no intensive care. Uncertainty around the results was characterized using one-way sensitivity analyses and a multivariate probabilistic sensitivity analysis. In the base-case analysis, neonatal intensive care for infants born at 24–26, 27–29, and 30–33 weeks gestational age prolonged life expectancy by 28, 43, and 34 years and averted 9, 15, and 12 DALYs, at incremental costs per infant of US$11,400, US$9,500, and US$3,000, respectively, compared to an alternative of no intensive care. The ICERs of neonatal intensive care at 24–26, 27–29, and 30–33 weeks were US$1,200, US$650, and US$240, per DALY averted, respectively. The findings were robust to variation in parameter values over wide ranges in sensitivity analyses.

**Conclusions:**

Incremental cost-effectiveness ratios for neonatal intensive care imply very high value for money on the basis of conventional benchmarks for cost-effectiveness analysis.

*Please see later in the article for the Editors' Summary*

## Abstract 2

Joshua Salomon and colleagues performed a cost-effectiveness analysis using health and economic outcomes following preterm birth in Mexico and showed that neonatal intensive care provided high value for the money in this setting.

## Editors' Summary

**Background:**

Most pregnancies last about 40 weeks but increasing numbers of babies are being born preterm, before they reach 37 weeks of gestation (the period during which a baby develops in its mother). In developed countries and some middle-income countries such as Mexico, improvements in the care of newborn babies (neonatal intensive care) mean that more preterm babies survive now than in the past. Nevertheless, preterm birth is still a major cause of infant death worldwide that challenges attainment of Target 5 of Millennium Development Goal 4—the reduction of the global under-five mortality rate by two-thirds of the 1990 rate by 2015 (the Millennium Development Goals, which were agreed by world leaders in 2000, aim to reduce world poverty). Furthermore, many preterm babies who survive have long-term health problems and disabilities such as cerebral palsy, deafness, or learning difficulties. The severity of these disabilities and their long-term costs to families and to society depend on the baby's degree of prematurity.

**Why Was This Study Done?:**

Mexico recently reformed its health system in an effort to improve access to care, particularly for the poorest sections of its population, and to improve the quality of its health care. The central component of this health care reform is the System of Social Protection of Health (SSPH). The SSPH contains a family health insurance program—Seguro Popular—that aims to provide the 50 million uninsured people living in Mexico with free access to an explicit set of health care interventions. As with any insurance program, decisions have to be made about which interventions Seguro Poplar should cover. Should neonatal intensive care be covered, for example? Do the benefits of this intervention (increased survival of babies) outweigh the costs of neonatal care and of long-term care for survivors with disabilities? In other words, is neonatal intensive care cost-effective? In this study, the researchers investigate this question by estimating the clinical benefits, costs, and cost-effectiveness of neonatal intensive care in Mexico.

**What Did the Researchers Do and Find?:**

The researchers built a decision analytic model, a mathematical model that combines evidence on the outcomes and costs of alternative treatments to help inform decisions about health care policy. They gathered data about the health outcomes of preterm births in Mexico from registers of births and deaths and from hospital discharge databases, and estimated the costs of neonatal intensive care and long-term care for disabled survivors using data from the Mexican Ministry of Health and the World Health Organization. They then applied their model, which estimates changes in parameters such as life expectancy, lifetime costs, disability-adjusted life years (DALYs; one DALY represents the loss of a year of healthy life), and incremental cost-effectiveness ratios (ICERs; the additional cost expended for each DALY averted) for neonatal intensive care compared to no intensive care, to a group of 2 million infants. Neonatal intensive care for infants born at 24–26, 27–29, and 30–33 weeks gestation prolonged life expectancy by 28, 43, and 34 years and averted 9, 15, and 12 DALYs at incremental costs of US$11,000, US$10,000, and US$3000, respectively, compared to no intensive care. The ICERs of neonatal intensive care for babies born at these times were US$1200, US$700, and US$300 per DALY averted, respectively.

**What Do These Findings Mean?:**

Interventions with ICERs of less than a country's per capita gross domestic product (GDP) are highly cost-effective; those with ICERs of 1–3 times the per capita GDP are potentially cost-effective. Mexico's per capita GDP in 2005 was approximately US$8,200. Thus, neonatal intensive care could provide exceptional value for money in Mexico (and maybe in other middle-income countries), even for very premature babies. The accuracy of these findings inevitably depends on the assumptions used to build the decision analytic model and on the accuracy of the data fed into it, but the findings were little changed by a wide range of alterations that the researchers made to the model. Importantly, however, this cost-effectiveness analysis focuses on health and economic consequences of different intervention choices, and does not capture all aspects of well-being. Decisions regarding neonatal intensive care will need to be based on a full consideration of all relevant factors, including ethical issues, and cost-effectiveness analyses should continue to be updated as new data emerge on health outcomes and costs associated with neonatal intensive care.

**Additional Information:**

Please access these Web sites via the online version of this summary at http://dx.doi.org/10.1371/journal.pmed.1000379.

- The March of Dimes, a nonprofit organization for pregnancy and baby health, provides information on preterm birth (in English and Spanish)
- The Nemours Foundation, another nonprofit organization for child health, also provides information on premature babies (in English and Spanish)
- MedlinePlus provides links to other information on premature babies (in English and Spanish)
- The United Nations Childrens Fund (UNICEF) works for children's rights, survival, development and protection around the world; it provides information on Millennium Development Goal 4 and its Childinfo website provides detailed statistics about child survival and health (some information in several languages)
- A *PLoS Medicine* Policy Forum by Núria Homedes and Antonio Ugalde discusses health care reforms in Mexico

## Introduction

Neonatal intensive care has dramatically improved the survival of preterm babies [1],[2]. However, as the borders of viability have been pushed to ever lower gestational ages (GAs), the costs of care have risen dramatically [3]–[6]. Furthermore, survival of the youngest patients frequently is accompanied by significant morbidity, placing substantial resource demands on patients, families, and society [7]–[10]. Middle-income countries such as Mexico have made significant progress in improving neonatal survival over the last decades. Nevertheless, elevated mortality rates for preterm infants persist in developing countries, compared to those in high-income countries [11].

Since the year 2000, Mexico has undertaken a comprehensive health system reform in order to improve access to care, equity, quality, and fairness of financing [12]. This reform focused on the 50 million uninsured Mexicans and established in 2004 the System of Social Protection in Health (SSPH). The SSPH contains a subsidized insurance-based component, Popular Health Insurance (*Seguro Popular*), which offers free access to an explicit set of health care interventions. The selection of interventions has been guided in part by evidence on the health and economic consequences of a wide array of candidate interventions, including explicit consideration of cost-effectiveness [13].

This study, undertaken as part of the process for selecting interventions to be included in the insurance benefits package, examined the clinical outcomes, lifetime costs, and cost-effectiveness of neonatal intensive care in Mexico.

## Methods

### Analytic Overview

**Figure 1:**
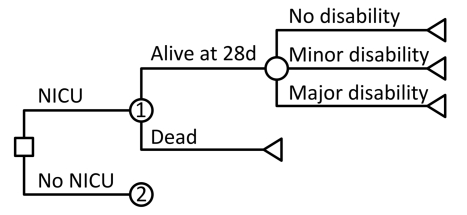
Schematic of decision tree model for outcomes during the neonatal period, with or without neonatal intensive care. Square indicates decision node, circles indicate chance nodes, and triangles indicate endpoints. Node 2 has the same structure as Node 1 but has different probabilities governing outcomes at each branch. Long-term outcomes for the endpoints of no disability, minor disability, and major disability are determined using a life table approach incorporating age-specific death rates defined for each disability category.

We developed a decision analytic model of health and economic outcomes following preterm birth in order to simulate the short-term and long-term consequences of neonatal intensive care compared to a counter-factual of no neonatal intensive care in Mexico. Analyses were stratified by three categories of GA at birth: 24–26 wk, 27–29 wk, and 30–33 wk. For each GA group, we used a decision tree to model outcomes during the neonatal period, with or without neonatal intensive care. These outcomes included mortality, survival to 28 d with no disability, or survival with either major or minor disability (Figure 1). Long-term outcomes among those surviving to the end of the neonatal period were modeled using a life table approach, with age-specific mortality rates dependent on long-term disability category. Outcomes were analyzed for the entire remaining lifetimes of the individuals.

Following guidelines from the World Health Organization (WHO) on conducting cost-effectiveness analyses [14], we adopted a societal perspective for measuring costs and health outcomes. Health outcomes were summarized in terms of life expectancy, disability-free life expectancy, and disability-adjusted life years (DALYs). Time costs were not included in the analysis under the assumption that these costs were relatively small in comparison to the costs of health care that were captured in the analysis. Costs were expressed as 2005 US dollars. Prices from earlier years were converted into 2005 units using gross domestic product (GDP) deflators. Prices available in Mexican pesos were first deflated to 2005 values and then converted into US dollars. Costs and DALYs were discounted at a rate of 3% per annum.

### Data on Mortality

Key parameters regarding health outcomes following preterm birth with or without neonatal intensive care were derived from Mexican vital registration and hospital discharge databases, supplemented with studies from the published medical literature, as needed. Because some previous studies have reported results stratified by birthweight rather than GA, in these cases we mapped from 500–749 g, 750–999 g, and 1,000–1,500 g birthweights to the GA groups 24–26, 27–29, and 30–33 wk, respectively. This mapping implied a weight-for-gestational-age around the 25th percentile on high-income national growth curves [15], which we intended to reflect the higher proportion of infants born small for GA in Mexico [16]. For example, the median weight for an infant born at 28 wk on a standard growth chart is about 1,200 g [15], but we applied outcomes from this group to the 30–33-wk GA group in the model.

Model input parameters relating to neonatal mortality are shown in Table 1. Neonatal mortality rates by GA were derived from hospital discharge records provided by the Mexican Ministry of Health. We pooled data from the years 2000 to 2005, which provided information on 90,526 births in the GA groups included in this study. Since neonatal intensive care units (NICUs) are the current standard of care in Mexico for treating preterm infants, we assumed that hospital discharge records provide information on neonatal mortality rates under full coverage of treatment. We derived estimates of neonatal mortality in the absence of intensive care from a meta-analysis of historical data on outcomes for very-low-birthweight infants in industrialized countries [17] and details from United States vital statistics on infant mortality during the era prior to the introduction of neonatal intensive care [18].

**Table 1:** Natural history and clinical variables, base-case values.

Variable	Values by GA (wk)		
	24–26	27–29	30–33
**Percent neonatal mortality, with neonatal intensive care** a	53	26	10
**Percent neonatal mortality, without neonatal intensive care** [17]	94	87	55
**Percent long-term morbidity, with neonatal intensive care** [22]–[26]			
Minor disability	38	32	12
Major disability	25	19	16
**Percent long-term morbidity, without neonatal intensive care** [17]			
Minor disability	46	46	25
Major disability	38	38	37
**Relative risks of mortality, major disability, by age group** [20],[21]			
0–9 y	24.6	24.6	24.6
10–19 y	8.5	8.5	8.5
20–39 y	3.6	3.6	3.6
40–69 y	2.2	2.2	2.2
70+y	1.5	1.5	1.5

Ranges used in sensitivity analyses, and details on the derivation of parameter values, are provided in Table S1 and Text S1.

a Computed from national hospital discharge database provided by Mexican Ministry of Health.

Postneonatal mortality rates by age were based on demographic estimates from the Consejo Nacional de Población in Mexico [19]. These rates were assumed to apply to individuals in the “no disability” and “minor disability” categories in the base-case analysis, although we conducted a sensitivity analysis that included elevated mortality among those with minor disabilities. For those with major disability, relative risks of mortality by age were derived from administrative databases recording outcomes among patients in the United States receiving services for intellectual impairment or cerebral palsy [20],[21], and multiplied by the Mexican age-specific mortality rates in the model. Further details on derivation of specific model parameters are included in Text S1.

### Data on Morbidity

In line with much of the empirical literature on morbidity following preterm birth, we distinguished two broad categories of disability. “Major disability” included cerebral palsy, moderate to severe intellectual impairment (defined as IQ<−2 standard deviations relative to normal birthweight controls), blindness, and deafness. “Minor disability” included learning difficulties, borderline to low average IQ (between −1 and −2 standard deviations), attention-deficit hyperactivity disorder (ADHD), unilateral or minor vision or hearing impairments, and persistent neuromotor abnormalities. For outcomes with neonatal intensive care, we derived probabilities of major or minor disability from meta-analyses, systematic reviews, and multiple-cohort studies on outcomes among low birthweight and preterm infants [22]–[26]. Outcomes were highly consistent over time and across studies for major disability, particularly for very low birthweight or very preterm infants. Estimates on the frequency of minor disability were more uncertain, in part due to lack of comparability in study designs and definitions of outcomes. Morbidity assumptions in the absence of neonatal intensive care were based on a previous meta-analysis of historical data on mortality and morbidity in industrialized countries [17]. Text S1 provides further details on the estimation of key probabilities governing morbidity outcomes in the model.

### Disability Weights

We derived average disability weights for major and minor disability on the basis of a previous study that elicited standard gamble utility values from parents for a wide range of pediatric outcomes [27]. Weights for the broad disability categories in our model were computed as frequency-weighted averages of the published weights for varying severity levels of intellectual impairments, cerebral palsy, vision and hearing impairments, and attention-deficit hyperactivity disorder (see Text S1 for details on the calculations). To assess the potential implications of uncertainty around these values on our results, we examined wide ranges around these point estimates in sensitivity analyses. The weights for specific disability categories were multiplied by regional, age-specific background disability weights presented by WHO [28].

### Data on Resource Use and Costs

Our model included medical costs accrued from the initial hospitalization, short-term costs related to rehospitalization, and long-term costs relating to health care for neurodevelopmental impairments. We used an ingredients approach to costing, by which the quantities of inputs (Table 2) that are used in delivering a particular service or intervention are multiplied by their unit prices (Table 3) to obtain total costs.

**Table 2:** Resource quantities, base-case values.

Variable	Values by GA (wk)		
	24–26	27–29	30–33
**Initial hospitalization** [29],[30]			
Days in hospital, survivors	116	86	49
Days in hospital, deaths	16	24	21
Proportion ventilated days (%)	47	31	17
**Nosocomial infections** [36]–[38]			
Percent probability of infection	93	52	41
Percent relative increase in costs for infants with infection (compared to infants without)	11	11	11
**Rehospitalization** [39]			
Days in hospital	7.4	4.0	2.6
**Surfactant** [33]–[35]			
Full doses per patient	1.0	0.3	0.4
**Percent probabilities of surgery** [30]			
Retinopathy of prematurity laser surgery	19	2	0.2
Ventriculo-peritoneal shunt placement	2	1	0.1
Patent ductus arteriosus ligation	31	11	3
Surgery for necrotizing enterocolitis	8	5	3

Ranges used in sensitivity analyses are described in Text S1 and Table S1.

**Table 3:** Resource unit costs (2005 US$), base-case values.

Resource Unit	Cost
**Ventilated hospital bed-day ** [**31**]	83
**Nonventilated hospital bed-day ** [**31**]	60
**Surfactant, per full dose** a	272
**Rehospitalization bed-day ** [**31**]	83
**Retinopathy of prematurity laser surgery** b	714
**Ventriculo-peritoneal shunt placement** b	1,710
**Patent ductus arteriosus ligation** b	6,250
**Surgery for necrotizing enterocolitis** b	1,450
**Long-term costs of disability, per person per year, by age (minor/major) ** [**40**],[41****]	
0–5 y	853/1,893
6–17 y	229/670
18–25 y	54/491
26–35 y	11/955
36–64 y	43/1,776
65+y	43/1,620

All unit costs were halved and doubled in sensitivity analyses.

a 2005 drug price list, Instituto Mexicano del Seguro Social de Mexico.

b 2004 charge list, Hospital Infantil de México Federico Gómez.

Direct costs associated with initial hospitalization were itemized into two main categories, general hospital costs and costs associated with neonatal-specific procedures/complications. Assumptions on the total number of hospital bed-days expected for each GA group were derived from registry data from the US National Institute of Child Health and Human Development Neonatal Research Network [29], distinguishing those who survived to discharge from those who died during the initial hospitalization. In order to accommodate different costs for ventilated versus nonventilated hospital bed days, we estimated the percentage of bed-days that were ventilated from a population-based national study of Canadian NICUs [30]. Unit prices per hospital bed-day were obtained from the WHO CHOICE database [31]. The CHOICE project estimates country-specific costs for a range of health services on the basis of an econometric analysis of multinational datasets on costs, using a small number of predictor variables including gross national income per capita. For ventilated NICU days, we applied the CHOICE estimate for Mexico for a bed-day in a tertiary-level hospital; for nonventilated bed-days we applied the CHOICE estimate for a secondary-level hospital.

In addition to general hospital fees, we accounted for resource use specific to neonatal intensive care, including provision of surfactant and surgeries commonly performed on preterm infants. Zupancic and colleagues [32] identified these costs as the largest nonpersonnel-related contributors to daily cost projections. We estimated the average number of doses of surfactant on the basis of a systematic review of surfactant therapy [33], with the number of doses for infants in the 24–26- and 27–29-wk groups derived from the OSIRIS trial [34]. The number of doses in the group above 30-wk GA was derived from a US study of moderately preterm infants [35], and we assumed that half of all infants would receive a second dose of surfactant. We also assumed that the required dose size for infants born at less than 30 wk GA was half that for infants born at 30–33 wk. The per-dose price for surfactant in Mexico was derived from the 2005 drug price list for the Instituto Mexicano del Seguro Social, which is the largest social insurance agency in Mexico.

We estimated the probabilities of receiving surgical interventions from a previous study in Canada [30]. Surgeries included patent ductus arteriosus ligation, ventriculo-peritoneal shunt placement, retinopathy of prematurity laser surgery, and surgery for necrotizing enterocolitis. Prices for each of these surgeries were obtained from the Hospital Infantil de México, the second largest pediatrics public hospital in the country, and a leading provider of neonatal intensive care under System of Social Protection in Health (SSPH).

Finally, we accounted for additional costs of the initial hospitalization related to health care-associated infections because of their high prevalence in middle-income country settings and their significant effect on resource utilization. Rates of infection were derived from two Brazilian studies [36],[37]. The incremental costs associated with these infections was were expressed as a percentage of the baseline cost of the initial hospitalization, which we estimated at 11% based on an analysis of nosocomial infections among surviving preterm infants from 17 North American hospitals [38].

Following the initial discharge, we estimated additional short-term medical costs relating to the rehospitalization of NICU survivors. Underwood [39] documented all-cause readmissions among preterm infants in California during the first year of life between 1992 and 2000. For each GA group, we derived average days of hospitalization by multiplying the average number of rehospitalizations per person by the average days per rehospitalization. For rehospitalizations we applied the tertiary-level bed-day estimate for Mexico from the CHOICE database.

We approximated long-term costs of disability by adapting estimates from a previous study on the costs of developmental impairments in the United States [40],[41]. The study estimated annual, per-person, age-specific costs for intellectual impairments, cerebral palsy, hearing loss, and vision impairment. We used the direct medical cost estimates from the study, which included physician visits, prescription medications, hospital inpatient stays, assistive devices, therapy, and rehabilitation (for persons aged <18 y), and long-term care (for persons aged 18–76 y), and rescaled these estimates using the ratio of Mexican GDP per capita to United States GDP per capita in 2005. These annual age-specific cost estimates were then applied in the life table model to compute discounted lifetime costs for persons in each disability category.

### Analysis

#### Base-case analysis

We estimated population-level neonatal outcomes in scenarios with and without neonatal intensive care in Mexico, for a birth cohort of around 2 million infants. We computed long-term, individual-level outcomes stratified by GA group, including life expectancy, disability-free life expectancy, DALYs, and total lifetime costs, with or without neonatal intensive care. We then calculated an incremental cost-effectiveness ratio (ICER) expressed as the difference in costs between the NICU and no-NICU scenarios (discounted at 3% per year), divided by the difference in DALYs in the two scenarios (also discounted at 3%). Following the standard benchmarks proposed in international work on cost-effectiveness, we compared the ICER to thresholds for cost-effectiveness defined in reference to the GDP per capita in Mexico. Interventions are considered to be highly cost-effective when they have ICERs that fall below the per capita GDP, and are regarded as being potentially cost-effective if they have ICERs between one and three times per capita GDP [42].

#### Sensitivity analyses

We conducted a series of univariate sensitivity analyses that varied each model input between upper and lower bounds reflecting uncertainty around the base-case parameter values (see Table S1 and Text S1 for details). We also performed a Monte Carlo probabilistic sensitivity analysis to assess the joint effects of uncertainty around all input parameters simultaneously (Text S1) [43]. In addition to these standard univariate and multivariate analyses, we conducted several further sensitivity analyses that were designed to impose deliberate bias against neonatal intensive care, for example by combining high estimates of mortality and morbidity associated with neonatal intensive care with high estimates of the costs of providing this care.

The model was implemented in Microsoft Excel 2010. Random variables for the Monte Carlo simulations were generated using Stata 11.

## Results

### Base Case

#### Population health outcomes

Table 4 shows the expected population health outcomes during the neonatal period, in a birth cohort of approximately 2 million infants, with and without neonatal intensive care. Across all GA groups combined, neonatal intensive care averts an estimated 20 thousand deaths but results in a net increase of 2,500 additional cases of minor or major disability. This latter increase is driven by higher absolute numbers of disabilities in the two younger GA groups, for which lower conditional probabilities of disability among survivors are more than offset by substantially increased survivorship. Overall, the greatest benefits of neonatal care, in both relative and absolute terms, are realized among the 30–33-wk GA group, which constitutes 70% of all preterm live births in Mexico.

**Table 4:** Population health outcomes during the neonatal period, with or without neonatal intensive care (thousands).

Outcome	GA (wk)			All
	24–26	27–29	30–33	
Number of births	4.6	8.2	29.1	42.0
***With neonatal intensive care***				
Neonatal deaths	2.5	2.1	2.9	7.5
Survival with no disability	0.8	3.0	18.9	22.7
Survival with minor disability	0.8	2.0	3.1	5.9
Survival with major disability	0.5	1.2	4.2	5.9
***Without neonatal intensive care***				
Neonatal deaths	4.3	7.2	16.0	27.5
Survival with no disability	<0.1	0.2	5.0	5.2
Survival with minor disability	0.1	0.5	3.3	3.9
Survival with major disability	0.1	0.4	4.9	5.4

#### Costs

Table 5 displays the estimated costs of neonatal intensive care by broad category. The largest contributors to overall care costs included the ventilated and nonventilated bed-day costs for the initial hospital stay, as well as long-term medical costs associated with chronic disabilities. As expected, costs are inversely related to GA, with the youngest group of infants requiring more resources than infants of at least 30 wk GA in most major cost categories, especially costs of surgery.

**Table 5:** Cost results per infant (2005 US$, thousands).

Cost Categories by Scenario	GA (wk)		
	24–26	27–29	30–33
***With neonatal intensive care***			
Initial hospitalization			
Ventilated days	2.5	1.8	0.7
Nonventilated days	2.0	2.9	2.3
Surfactant	0.3	0.1	0.1
Surgery cost	2.2	0.8	0.2
Infection cost	0.7	0.3	0.1
Rehospitalization	0.3	0.2	0.2
Long-term costs of disability	4.0	4.9	4.1
**Total (discounted** a **) lifetime costs**	**12.0**	**11.1**	**7.7**
***Without neonatal intensive care***			
Rehospitalization	<0.1	<0.1	0.1
Long-term costs of disability	0.5	1.6	4.6
** Total (discounted** a **) lifetime costs**	**0.6**	**1.6**	**4.7**

a Costs are discounted at a rate of 3% per year.

#### Life expectancy and disability-free life expectancy

In the absence of neonatal intensive care, we estimated average life expectancy to be 4, 8, and 28 y for infants born at 24–26, 27–29, and 30–33 wk GA, respectively. With NICU, corresponding life expectancies were 32, 51, and 63 y, implying gains of 28, 43, and 34 y. Gains in disability-free life expectancy attributable to neonatal intensive care would be smaller but still substantial for the two lower GA groups (12 and 26 y for 24–26 and 27–29 wk, respectively), and slightly greater for the 30–33-wk group (36 y).

#### Cost-effectiveness

Table 6 summarizes the results of our base-case analysis. Providing neonatal intensive care yields gains in disability-adjusted life expectancy (which include adjustments for decreased health status and discounting at a rate of 3% per year) of 9, 15, and 12 y, for infants born at 24–26, 27–29, and 30–33 wk GA, respectively, at incremental costs per infant of US$11,400, US$9,500, and US$3,000. Incremental cost-effectiveness ratios for NICU compared to no-NICU by GA were approximately US$1,200 per DALY for the 24–26-wk group, US$650 per DALY for 27–29 wk, and US$240 per DALY for 30–33 wk. Based on typical benchmarks for international cost-effectiveness analysis, defined in reference to the national GDP per capita (which was approximately US$8,200 in Mexico in 2005 [44]), neonatal intensive care at all GA groups would be regarded as exceptional value for money, costing only a fraction of the per capita national income for each year of healthy life that it saves.

**Table 6:** Changes per person in life expectancy, disability-free life expectancy, DALYs, costs, and ICERs for neonatal intensive care compared to no neonatal intensive care, base-case analysis.

GA Group	Change in LE (y)	Change in DFLE (y)	DALYs Averted (y)	Incremental Costs (2005 US$)	ICER (US$/DALY)
24–26 wk	28	12	9	11,400	1,230
27–29 wk	43	26	15	9,500	650
30–33 wk	34	36	12	3,000	240

Life expectancy (LE) and disability-free life expectancy (DFLE) are undiscounted. Costs and DALYs are discounted at a rate of 3% per year.

### Sensitivity Analysis

In one-way analyses that varied each parameter across a range of values, cost-effectiveness ratios were minimally sensitive to changes in input values (Figure S1; Table S2; Text S1). None of the univariate analyses produced cost-effectiveness ratios exceeding US$1,800 per DALY averted in the 24–26-wk group, US$900 per DALY in the 27–29-wk group, or US$500 per DALY in the 30–33-wk group. Even these maximum values fall well below the threshold of national GDP per capita.

Notwithstanding the robustness of the conclusions to variation in input values, interesting differences were observed in the relative importance of different inputs across GA groups (Figure S1). In the 24–26-wk group, results were most sensitive to assumptions about neonatal mortality, and relatively insensitive to assumptions about disability. This finding is consistent with the high mortality rates overall in this youngest group, which reduce the importance of morbidity outcomes. Conversely, the results in the 30–33-wk group were most sensitive to assumptions about major disability, in line with the better survivorship outcomes in this group. Across all three GA groups, costs of ventilated and nonventilated bed-days were consistently among the most important variables as drivers of the cost-effectiveness results.

On the basis of a set of multivariate Monte Carlo simulations in which we jointly varied all input parameters, we constructed cost-effectiveness acceptability curves to consider the likelihood that NICU would be cost-effective under different thresholds for societal willingness to pay for an additional year of healthy life (Figure S2). If society were willing to pay up to the average per capita income for each year of life gained, the results suggest essentially no uncertainty in the conclusion that neonatal intensive care would be cost-effective in all GA groups. Even at a threshold of only US$1,000 (representing only 12% of per capita GDP), the probabilities that neonatal intensive would be regarded as cost-effective are 10%, 95%, and 100% in the 24–26-wk, 27–29-wk, and 30–33-wk groups, respectively. At twice this value (or approximately one-quarter of per capita GDP), the probabilities are 92%, 100%, and 100%.

Given the very attractive overall cost-effectiveness ratios and the relative insensitivity of these results to variation in individual parameter values or joint variation across all inputs, we considered a further set of sensitivity analyses intended to impose a strong bias against neonatal intensive care. First, we assumed that NICU would confer only survivorship benefits (by raising the morbidity probabilities in the NICU strategy to be equal to those in the no-NICU strategy). This change resulted in cost-effectiveness ratios of US$1,600, US$1,100, and US$880 per DALY across the three GA groups. Next, we preserved this assumption of no morbidity benefits and also raised NICU mortality probabilities to the upper bounds of their ranges, which were 75%, 51%, and 14%, compared to the base-case values of 53%, 26%, and 10%, reflecting relative increases of around 40% for the youngest and oldest groups, and 100% for the middle group. The resulting cost-effectiveness ratios were US$2,200, US$1,300, and US$900 per DALY. Finally, we maintained all of the unfavorable mortality and morbidity assumptions described above, and also doubled all unit costs in the model, which resulted in cost-effectiveness ratios of US$4,400, US$2,600, and US$1,800 across the groups. Overall, these results confirm that neonatal intensive care appears highly cost-effective even under conditions that are dramatically less favorable than our base-case assumptions regarding both health benefits and costs associated with neonatal intensive care.

## Discussion

In this study we undertook a comprehensive examination of the costs and health benefits associated with providing neonatal intensive care to preterm infants in Mexico. Contrary to the widely held belief that neonatal intensive care imposes excessive resource demands relative to the benefit it confers, we found that it offers exceptional value for money even in the youngest GA group. Our conclusions were robust to all variations from base-case assumptions in the model.

For middle-income countries, more widespread access to neonatal intensive care services could be an important component of efforts to achieve Millennium Development Goal 4—to reduce child mortality by two-thirds by 2015 [45]. Every year it is estimated that 4 million babies die in the first month of life, and 28% of those die from preterm birth [46]. Our study suggests that neonatal intensive care, despite being regarded by some as prohibitively expensive, provides high returns in health for the amount of resources it consumes. Many highly effective prenatal and postnatal care practices are affordable. Antenatal steroids and maternal antibiotics for prolonged preterm rupture of membranes can yield substantial improvements in preterm survival and reductions in morbidity [47]. Postnatal use of low-cost interventions—such as ventilation with bubble continuous positive airway pressure, temperature support, blended oxygen, kangaroo care, early initiation of breast milk feeding, and infection control measures that include family members as stakeholders—have been shown to be effective [48]–[53].

Many of the assumptions in our cost-effectiveness model were deliberately biased against neonatal intensive care. For example, we assumed a relatively modest reduction in rates of disability in the 30–33-wk GA group compared to those to those in the 27–29-wk group, despite some data from high-income countries suggesting that rates are significantly lower in older preterm infants. Likewise, our mortality estimates for the comparator scenario of no neonatal intensive care may be too low, which would result in an underestimate of the health benefits of intensive care. In general, when forced to reconcile several conflicting data sources, we elected assumptions that would be most unfavorable toward neonatal intensive care.

In sensitivity analyses, even a worst-case scenario in which NICU conferred substantially reduced survivorship benefits and no benefits in terms of averting disability among survivors, and all unit costs were twice as high as their base-case estimates, cost-effectiveness ratios for neonatal care in all GA groups remained well below the typical benchmark for high-value interventions. Despite the favorable cost-effectiveness estimates, however, we recognize that decisions regarding the care of extremely preterm infants are complicated by a range of critical considerations that can place significant burdens on families and necessitate substantial financial investments in health and educational services. Cost-effectiveness information must be regarded as only one of several important factors to consider in making individual decisions and designing health policies. It is essential that decisions be informed by an array of perspectives, including ethical debate.

This study indicates that neonatal intensive care adds a significant number of disability-free years to the population of preterm infants. Nevertheless, with increasing survival the absolute burden of disability is likely to rise, at least in some groups. In our model, we find that for infants born earlier than 30 wk GA, neonatal intensive care is expected to increase the absolute number of cases of disability. While some of the specific impairments are relatively minor (behavioral and learning problems or attention-deficit hyperactivity disorder), some infants will be profoundly disabled, for example with nonambulatory cerebral palsy, blindness, deafness, or severe intellectual impairment.

Severe disability can place a heavy emotional and financial burden on patients, families, and society. In line with standard guidance for economic evaluation, the perspective of our cost-effectiveness analysis includes all health and economic outcomes associated with health care intervention, but omits other dimensions of well-being that may be important to individual decision-making. Of note, a major study on parent preferences over pediatric impairments [27]—which we used to define health-state valuations in this study—suggested that families typically have strong preferences for life even with disability. In addition, as a group, extremely preterm infants appear to enjoy similar health-related quality of life upon reaching young adulthood, compared with normal birthweight peers [54],[55]. Health care professionals have also been shown to vastly underestimate quality of life for infants with disability [56]. On the other hand, it is worth noting that another study reported substantially lower health-related quality of life among a German preterm birth cohort compared with Canadian and Dutch cohorts, suggesting that significant differences may exist between countries [57]. While it is important to realize that in Mexico neonatal intensive care is already established practice, the substantial societal burden associated with extreme prematurity warrants systematic evaluation and supportive family intervention.

Our results are compatible with previous analyses conducted in high-income nations, which have found neonatal intensive care to be cost-effective [5],[6],[58]. To our knowledge there is only one published economic evaluation of neonatal intensive care in a middle-income country, undertaken in Malaysia [59]. In contrast to our study, that analysis focused on health effects and costs during the initial hospitalization only and yielded a cost-effectiveness ratio of US$4,200 (in 2004 US$ adjusted for inflation using GDP deflators [60]) for infants between 1,000–1,500 g birthweight. This ratio falls below Malaysia's 2004 per capita income US$4,960 [44], which indicates high value for money.

We emphasize the importance of interpreting our results in light of the intended context of the study. Our purpose was not to provide a precise estimate of the cost-effectiveness of neonatal intensive care in Mexico. The required data for such an endeavor are simply not available. Rather, we aimed to inform the debate on funding for neonatal intensive care by synthesizing the best available evidence. We have attempted to maximize internal and external validity as well as generalizability. Whenever possible, we chose amongst available data sources with an interest in parsimony and transparency, while at the same time ensuring that the model would be based on defensible assumptions. We gave priority to local data wherever possible for both the base-case and sensitivity analyses. In the interest of generalizability, we compared neonatal intensive care to a “null” alternative as recommended in WHO guidelines [14]. Therefore, we hope that the results in this study will provide useful information in other middle-income countries facing similar funding decisions.

Nevertheless, there remains a considerable amount of uncertainty surrounding several of our model inputs. The most critical inputs in the model are the probabilities of mortality and morbidity and estimates of NICU costs. Our data for mortality reflect national experience among hospitalized infants; they may not capture the precise mortality risks faced by the currently uninsured population to whom health insurance expansion has been directed. It is also important to note that our results represent an overall estimate of cost-effectiveness of neonatal intensive care. There is likely significant variability in outcomes based on differences in population risk, socioeconomic status, and access to health care services. Addressing these disparities will require investments in the health care system (e.g., regionalized care and patient transport system, early intervention therapists, medical subspecialists) and other areas of the economy (e.g., transportation, labor market reform, etc.). Such investments are likely to occur over time and are beyond the scope of this analysis.

In terms of morbidity assumptions, data limitations required that we rely on information from high-income countries, which might contradict our approach to biasing assumptions against neonatal intensive care. Morbidity is possibly higher in Mexico than in high-income nations, and there have been reports of an epidemic of blindness among survivors of neonatal intensive care in middle-income countries [61],[62]. However, we believe the our choice of data inputs was appropriate for several reasons, including: (1) high rates of prenatal care in Mexico; (2) high probabilities that births occur in a health facility (93% by one estimate) [63]; (3) low frequency of low birthweight and very low birthweight deliveries [64]; (4) focus of health reform for 2004–2010 on 100% coverage of preterm newborns to reduce mortality rates among infants born at 30–34 wk [65]; (5) more favorable pregnancy outcomes among Mexican-American women born in Mexico compared to US-born Hispanics [66]; and (6) comparable neurodevelopmental outcomes at 6 y (compared to Doyle et al. [2]) in the only published recent study [67]. In addition, even an alternative assumption of no NICU-associated reductions in probabilities of disability for surviving infants preserved the conclusion that neonatal intensive care is highly cost-effective in the Mexican setting.

Our cost inputs are derived partly from estimates for Mexico from the WHO-CHOICE database, which may only approximate true economic costs in Mexico. These costs are predicted from an econometric model that uses a relatively small number of independent variables. It is therefore likely that some variation across countries will be underestimated in the modeled prices. Again, however, even a doubling in costs did not yield cost-effectiveness ratios that even approached the boundaries for high-value interventions, which provides reassurance that our conclusions are robust despite imprecision of cost estimates.

Lastly, our long-term cost estimates are based on a study conducted in the United States [40],[41]. This study includes costs for physician visits, hospital stays, assistive devices, therapy and rehabilitation, and long-term care. These specific cost elements are unlikely to extrapolate perfectly into practice in Mexico. On the other hand, other complications due to preterm birth, such as additional care required for chronic lung disease, are not captured. Overall, we believe that this data source represented a reasonable approximation of long-term costs in Mexico. As above, we note further that a doubling of these costs did not alter the finding that neonatal intensive care would be highly cost-effective.

In summary, our economic evaluation indicates that neonatal intensive care for preterm infants in Mexico is likely to be exceedingly cost-effective. While improving the survival of infants above 30 wk GA provides the greatest overall population health benefits, and at the highest value for money, intervention among all preterm infants above 24 wk GA should be considered as a cost-effective use of health care resources. As future research proceeds on interventions for neonatal intensive care, the societal value of these interventions should continue to be evaluated. Likewise, as new evidence accumulates on health outcomes and resource requirements associated with these interventions in a broader array of settings, questions about costs, benefits, and efficiency should be revisited in light of the best available evidence.

## Supporting Information

Figure S1 Results from univariate sensitivity analyses, by GA group.
(0.15 MB PDF)

Figure S2 Cost-effectiveness acceptability curves for neonatal intensive care compared to no neonatal intensive care, by GA group.
(0.03 MB PDF)

Table S1 Parameter ranges examined in sensitivity analyses.
(0.05 MB PDF)

Table S2 Results from univariate sensitivity analyses.
(0.06 MB PDF)

Text S1 Technical appendix. The supporting tables and figures are available as individual files (Figures S1 and S2 and Tables S1 and S2), but are also included here for ease of access.
(0.46 MB PDF)

## Abbreviations

DALY: disability-adjusted life years
GA: gestational age
GDP: gross domestic product
ICER: incremental cost-effectiveness ratio
NICU: neonatal intensive care unit
