# Supplementary figures and images for: Clinical Benefits, Costs, and Cost-Effectiveness of Neonatal Intensive Care in Mexico
Source: PLoS Med. 2010 Dec 14;7(12):e1000379. doi: 10.1371/journal.pmed.1000379 (PMC3001895; doi:10.1371/journal.pmed.1000379)

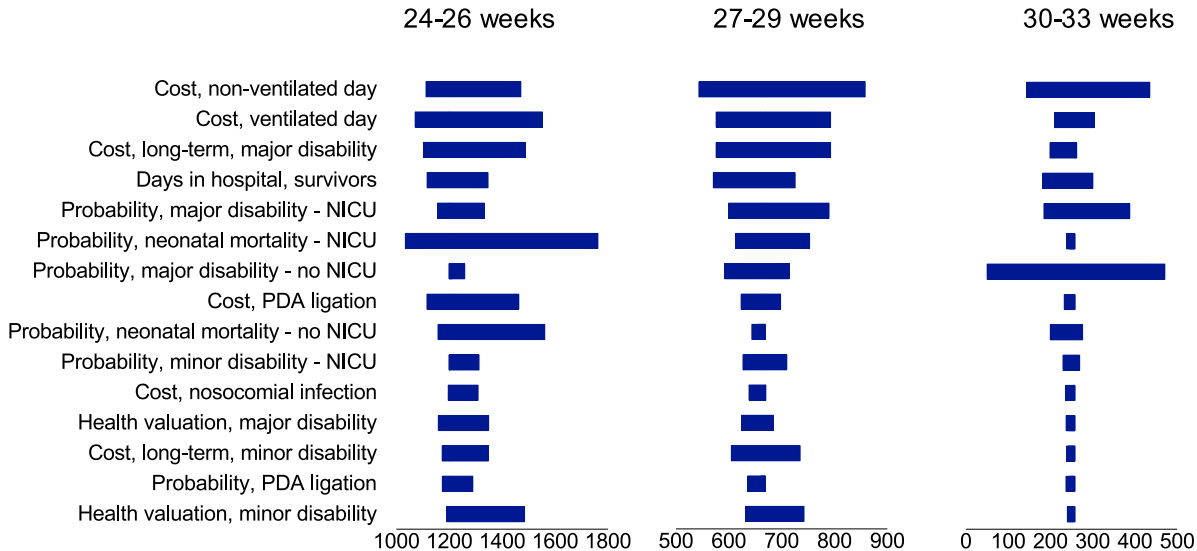

Incremental cost-effectiveness ratio for NICU compared to no NICU

Supplement: Figure S1 — Results from univariate sensitivity analyses, by GA group. (0.15 MB PDF) [file pmed.1000379.s001.pdf]

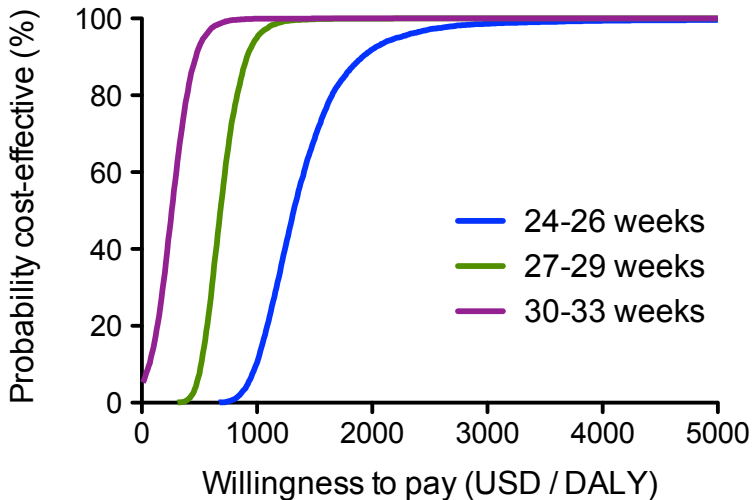

Supplement: Figure S2 — Cost-effectiveness acceptability curves for neonatal intensive care compared to no neonatal intensive care, by GA group. (0.03 MB PDF) [file pmed.1000379.s002.pdf]
