# Supplementary material for: Clinical Benefits, Costs, and Cost-Effectiveness of Neonatal Intensive Care in Mexico
Source: PLoS Med. 2010 Dec 14;7(12):e1000379. doi: 10.1371/journal.pmed.1000379 (PMC3001895; doi:10.1371/journal.pmed.1000379)
Supplement: Table S1 — Parameter ranges examined in sensitivity analyses. (0.05 MB PDF) [file pmed.1000379.s003.pdf]

Table S1. Parameter ranges examined in sensitivity analyses.

Parameter	Lower bound	Upper bound
**Neonatal mortality probabilities**		
With NICU, 24-26 weeks	0.23	0.75
With NICU, 27-29 weeks	0.08	0.51
With NICU, 30-33 weeks	0.03	0.14
Without NICU	BC x 0.85	BC x 1.16*
**Probabilities of long-term morbidity**		
Minor disability, with NICU	BC x 0.84	BC x 1.43†
Minor disability, without NICU	BC x 0.43	BC x 1.57†
Major disability, with NICU	BC x 0.84	BC x 1.43
Major disability, without NICU	BC x 0.43	BC x 1.57
**Relative risks of mortality**		
Minor disability	§	§
Major disability	BC x 0.75	BC x 1.25
**Health-state valuations**		
Minor disability	0.53	1.0
Major disability	0.19	1.0
**Initial hospitalization**		
Days in hospital, survivors	BC x 0.75	BC x 1.25
Days in hospital, deaths	BC x 0.75	BC x 1.25
Proportion ventilated days	BC x 0.75	BC x 1.25
**Nosocomial infection**		
Probability of infection	BC x 0.75	BC x 1.25*
Relative increase in costs	BC x 0.5	BC x 2
**Rehospitalization days**	BC x 0.75	BC x 1.25
**Surfactant doses**	BC x 0.75	BC x 1.25
**Probabilities of surgery**		
Retinopathy of prematurity	BC x 0.75	BC x 1.25
VP shunt	BC x 0.75	BC x 1.25
PDA ligation	BC x 0.75	BC x 1.25
Necrotizing enterocolitis	BC x 0.75	BC x 1.25
**Unit costs**		
Ventilated bed-day	BC x 0.5	BC x 2
Non-ventilated bed-day	BC x 0.5	BC x 2
Surfactant	BC x 0.5	BC x 2
Retinopathy of prematurity	BC x 0.5	BC x 2
VP shunt	BC x 0.5	BC x 2
PDA ligation	BC x 0.5	BC x 2
Necrotizing enterocolitis	BC x 0.5	BC x 2
Long-term costs, minor disability	BC x 0.5	BC x 2
Long-term costs, major disability	BC x 0.5	BC x 2

* Upper bound truncated at 0.99.
† Where probabilities of major and minor disability summed to greater than 1, probability of minor disability was truncated to constrain the sum to 1.
§ In the base-case analysis, we assumed that minor disability produced no excess mortality. In sensitivity analyses, the upper bound reflects an alternative assumption that the excess risk (RR – 1) for minor disability was half the base-case (RR – 1) for major disability. The lower bound was equal to the base case assumption that RR = 1.0.

Abbreviations: NICU – neonatal intensive care unit; GA – gestational age; BC – base-case value; VP – ventriculo-peritoneal; PDA – patent ductus arteriosus; RR – relative risk.
