# Supplementary material for: Clinical Benefits, Costs, and Cost-Effectiveness of Neonatal Intensive Care in Mexico
Source: PLoS Med. 2010 Dec 14;7(12):e1000379. doi: 10.1371/journal.pmed.1000379 (PMC3001895; doi:10.1371/journal.pmed.1000379)
Supplement: Table S2 — Results from univariate sensitivity analyses. (0.06 MB PDF) [file pmed.1000379.s004.pdf]

Table S2. Results from univariate sensitivity analyses.

	Incremental cost-effectiveness of NICU vs. no NICU*					
	24-26 weeks GA		27-29 weeks GA		30-33 weeks GA	
**Parameter**	**At lower bound**	**At upper bound**	**At lower bound**	**At upper bound**	**At lower bound**	**At upper bound**
**Neonatal mortality probabilities**						
With NICU	1,034	1,762	613	753	245	238
Without NICU	1,561	1,158	656	644	200	276
**Probabilities of long-term morbidity**						
Minor disability, with NICU	1,202	1,313	627	710	230	269
Minor disability, without NICU	1,245	1,222	664	636	272	214
Major disability, with NICU	1,156	1,333	600	790	185	388
Major disability, without NICU	1,259	1,210	714	592	471	51
**Relative risks of mortality**						
Minor disability†	1,232	1,311	648	672	241	241
Major disability	1,246	1,220	661	638	237	245
**Health-state valuations**						
Minor disability	1,485	1,190	742	632	240	241
Major disability	1,349	1,159	685	624	238	244
**Initial hospitalization**						
Days in hospital, survivors	1,117	1,347	571	726	182	301
Days in hospital, deaths	1,214	1,250	641	656	238	244
Proportion ventilated days	1,211	1,252	639	657	237	245
**Nosocomial infection**						
Probability of infection	1,213	1,237	643	654	238	244
Relative increase in costs	1,197	1,309	638	670	236	253
**Rehospitalization days**	1,225	1,239	645	652	239	243
**Surfactant doses**	1,223	1,240	647	650	239	243
**Probabilities of surgery**						
Retinopathy of prematurity	1,228	1,236	648	649	241	241
VP shunt	1,231	1,233	648	649	241	241
PDA ligation	1,174	1,290	636	661	237	245
Necrotizing enterocolitis	1,228	1,235	647	650	240	242
**Unit costs**						
Ventilated bed-day	1,072	1,552	576	793	210	304
Non-ventilated bed-day	1,112	1,471	544	858	144	435
Surfactant	1,215	1,266	645	655	237	250
Retinopathy of prematurity	1,224	1,248	648	649	241	241
VP shunt	1,230	1,236	648	650	241	241
PDA ligation	1,116	1,463	624	698	233	258
Necrotizing enterocolitis	1,225	1,246	646	654	239	245
Long-term costs, minor disability	1,174	1,348	605	735	242	239
Long-term costs, major disability	1,104	1,488	576	793	262	199

* 2005 US dollars per disability-adjusted life year
† Lower bound reflects base-case assumption that minor disability produces no excess mortality.

Abbreviations: NICU – neonatal intensive care unit; GA – gestational age; VP – ventriculo-peritoneal; PDA – patent ductus arteriosus.
