# Supplementary material for: Clinical Benefits, Costs, and Cost-Effectiveness of Neonatal Intensive Care in Mexico
Source: PLoS Med. 2010 Dec 14;7(12):e1000379. doi: 10.1371/journal.pmed.1000379 (PMC3001895; doi:10.1371/journal.pmed.1000379)

## Clinical Benefits, Costs and Cost-Effectiveness of Neonatal Intensive Care in Mexico

Jochen Profit, Diana Lee, John A Zupancic, LuAnn Papile, Cristina Gutierrez, Sue J Goldie, Eduardo Gonzalez-Pier, Joshua A Salomon

# Text S1. Technical Appendix

This appendix provides details on (1) the sources and derivation of base-case values and ranges for input parameters; and (2) methods and results for univariate and multivariate sensitivity analyses.

### Estimation of parameter values and ranges for health outcomes in the model

#### *Mortality with neonatal intensive care*

Neonatal mortality rates by gestational age were derived from hospital discharge records provided by the Mexican Ministry of Health. We pooled data from the years 2000 to 2005, which provided information on 90,526 births in the gestational age (GA) groups included in this study. Since neonatal intensive care units are the current standard of care in Mexico for treating preterm infants, we assumed that hospital discharge records provide information on neonatal mortality rates under full coverage of treatment. Lower bounds for mortality in the presence of neonatal intensive care for preterm infants were derived from population-based data from Norway and the Netherlands [1,2]. Upper bounds for mortality reflect the experience in a single Mexican tertiary care center [3].

#### *Mortality without neonatal intensive care*

We derived estimates of neonatal mortality in the absence of intensive care from a meta-analysis of historical data on outcomes for very-low-birthweight infants in industrialized countries [4] and details from United States vital statistics on infant mortality from the era prior to the introduction of neonatal intensive care [5]. Results from the two sources were highly consistent. The meta-analysis reported a mortality probability of 55% (95% confidence interval 47-64) for infants under 1500g, observed during the period 1947-65. The U.S. vital statistics for the 1950 birth cohort reported the same value for the birthweight group 1000-1500g, and reported 87% mortality in the <1000g group. In line we these results we used point estimates of 87% and 55% for the 27-29 week and 30-33 week GA groups, respectively. For the 24-26 week group there were no results reported, so we assumed that survival in this group would be only half as great as in the 27-29 week group, which yielded a point estimate of 94% mortality. Ranges around these point estimates were derived based on the confidence interval from the meta-analysis, assuming that the same relative distance to the lower and upper bound would apply to all groups.

#### *Morbidity with neonatal intensive care*

In line with much of the empirical literature on morbidity following premature birth, we distinguished two broad categories of disability. Major disability included cerebral palsy, moderate to severe intellectual impairment (defined as IQ <-2 standard deviations relative to normal birthweight controls), blindness and deafness. Minor disability included learning disabilities, borderline to low average IQ (between -1 and -2 standard deviations), attention-deficit hyperactivity disorder (ADHD), unilateral or minor vision or hearing impairments, and persistent neuromotor abnormalities. For outcomes with neonatal intensive care, we derived probabilities of major or minor disability from meta-analyses, systematic reviews and multiple-cohort studies on outcomes among low birthweight and premature infants [6,7,8,9,10]. Outcomes were highly consistent over time and across studies for major disability, particularly for very low birthweight or very premature infants. For example, studies by Escobar et al. [6] Lorenz [8], and Saigal & Doyle [10] estimated the probabilities of major disability among very low birthweight infants to be 25%, 24% and 26%, respectively. We used a point estimate of 25% for the probability of major disability in the lowest GA group, with a range defined by the 95% confidence interval reported by Escobar et al. (21-30%). Corresponding probabilities in older GA groups were based on the ratios observed in the Victorian Infant Collaborative Study Group [9] and in the review by Escobar et al., which indicated that disability probabilities decline by around 20-25% in moving from lower to higher birthweight categories. Estimates on the frequency of minor disability were more uncertain, in part due to lack of comparability in study designs and definitions of outcomes. We derived the probability of minor disability in each category by subtracting the point estimates of major disability from the estimates of all forms of morbidity reported by Taylor et al. [11] in their study on school-age outcomes for very low birthweight children. To cross validate these results, we compared them to reported estimates of minor disability in the Victorian study, and found them to be quite similar. For example, our estimate of 38% minor disability in the 24-26 week group matched closely the Roberts et al. [9] estimate of 37%. Likewise, in the middle GA group our estimate of 32% was similar to

the Roberts et al. estimate of 28%. Ranges for all probabilities of disability were assumed to match the relative distances below and above the point estimate reflected in the Escobar et al. review [6].

### *Morbidity without neonatal intensive care*

Morbidity assumptions in the absence of neonatal intensive care were based on a previous meta-analysis of historical data on mortality and morbidity in industrialized countries [4]. Probabilities of major and minor disability were reported for the birthweight category of <1500g in periods before the introduction of NICU (1947-1965) and the period of early introduction (1966-1970). We used the estimate from the former for the 30-33 week GA group in our model. For the 24-26 and 27-29 week GA groups, the only available referent was the outcome for infants <1000g from the period 1966-1970. We assumed that the observed trend in the probability of no disability for the <1500g group between the two periods would also pertain to the <1000g group, and we applied the resulting probabilities to both the 24-26 and 27-29 week groups in our model. Ranges around all of the disability probability estimates in the absence of neonatal intensive care were derived from the reported confidence interval for the <1500g group, which spanned +/- 57% of the point estimate for major disabilities and +/- 43% of the point estimate for minor disabilities.

### *Relative risks of post-neonatal mortality for persons with major or minor disability*

For those with major disability, relative risks (RR) of mortality by age were derived from administrative databases recording outcomes among patients in the United States receiving services for intellectual impairments or cerebral palsy [12,13]. Following the same approach described by Honeycutt and colleagues in their analysis of the economic costs of developmental impairments [14,15], we compared reported mortality by age among patients with intellectual impairments to U.S. life tables from the same period to derive relative mortality risks for intellectual impairments. For cerebral palsy, we obtained age-specific relative risks already reported in the prior study. Combined relative risks for the category of major disability were computed assuming a ratio of 1.7 to 1 for intellectual impairment relative to cerebral palsy, based on the observed ratio in a meta-analysis of outcomes in low birthweight infants [8]. In sensitivity analyses we considered a range around these relative risks spanning +/- 25%. For minor disability our base-case analysis assumed that there was no excess mortality, but in sensitivity analysis we considered a scenario in which the excess risk (RR – 1) for minor disability was half of the corresponding age-specific base-case value for major disability.

### *Disability weights*

We derived average disability weights for minor and major disability based on a previous study that elicited standard gamble utility values from parents for a wide range of pediatric outcomes [16]. Computations were done on a 'health-state valuation' scale, which is the inverse of the disability weight scale. The valuation for major disability was computed as a weighted average of the reported values for mild, moderate or severe intellectual impairments; mild, moderate or severe cerebral palsy; blindness; and deafness. Frequency weights on each condition were derived from their relative incidence estimates in the systematic review of low birthweight outcomes by Lorenz [8], in order to compute an average health-state valuation for a person with only one condition. To allow for worse health-state valuations in the presence of multiple conditions, we used the estimated proportion of people with more than one condition from the Lorenz study (43%) and incorporated an average value for a person with two conditions using a multiplicative model in the overall estimated value for major disability. To estimate a range around the valuation we undertook analogous calculations based on the lower and upper bounds of the confidence intervals for each condition. The resulting estimate for the health-state valuation of major disability was 0.66, with a range from 0.19 to 1.0. For minor disability, we used a simple unweighted average of the reported valuations for mild ADHD, mild hearing impairment, moderate hearing impairment, mild bilateral vision loss and severe ADHD. The resulting valuation and range were 0.92 and (0.53, 1.0). In the life table model the weights for specific disability categories were multiplied by regional, age-specific background disability weights presented by WHO in calculating disability-adjusted life years [17].

### Sensitivity analyses

We varied all parameters in univariate sensitivity analyses to consider uncertainties in health outcomes with and without neonatal intensive care, and costs of related healthcare services and other resource consumption associated with preterm birth. Data sources and assumptions used to define ranges around outcome parameters are described above, and the ranges are summarized in Table S1. For most resource quantities we considered ranges that spanned +/- 25% of the base-case values. Given substantial uncertainty around unit costs, we allowed for wider ranges around these parameters, subtracting 50% or adding 100% to the base-case values. In order to assess the joint effects of uncertainty around all input parameters simultaneously, we performed a multivariate, probabilistic sensitivity analysis based on 10,000 Monte Carlo simulations. We assumed that each parameter was an independent random variable with a distribution defined in reference to the low and high estimates used

in univariate sensitivity analyses (summarized in Table S1). For all parameters except unit costs, we assumed that each variable was characterized by a beta distribution, with mean value equal to the base-case value and standard error computed as (upper bound – lower bound) / (2 x 1.96). For unit costs, we assumed lognormal distributions consistent with the halving and doubling of base-case values at the lower and upper bounds. The specific beta and lognormal distributions used were consistent with an interpretation of the ranges in Table S1 as 95% uncertainty intervals.

Results from univariate sensitivity analyses are shown in Table S2 for all variables, and summarized as tornado diagrams in Figure S1. In Figure S1, results are shown for the 15 variables with the greatest impact on average across the 3 GA groups. They are ordered from top to bottom in terms of this average impact, which provides a simple visual indication of differences in the importance of variables across the different GA groups. Results of the probabilistic sensitivity analyses are summarized as cost-effectiveness acceptability curves (Figure S2), which show the probability that neonatal intensive care would be cost-effective compared to the counterfactual of no neonatal intensive care, under various thresholds representing societal willingness to pay to avert one disability-adjusted life year.

**References**


1. Markestad T, Kaaresen PI, Ronnestad A, Reigstad H, Lossius K, et al. (2005) Early death, morbidity, and need of treatment among extremely premature infants. Pediatrics 115: 1289-1298.
2. Stoelhorst GM, Rijken M, Martens SE, Brand R, den Ouden AL, et al. (2005) Changes in neonatology: comparison of two cohorts of very preterm infants (gestational age <32 weeks): the Project On Preterm and Small for Gestational Age Infants 1983 and the Leiden Follow-Up Project on Prematurity 1996-1997. Pediatrics 115: 396-405.
3. Osorno-Covarrubias L, Vela-Urtecho G, Davila-Velazquez J (2002) [Graphic representation of the neonatal mortality risk at a regional perinatal center in Merida, Yucatan, Mexico]. Salud Publica Mex 44: 345-348.
4. Lee KS, Kim BI, Khoshnood B, Hsieh HL, Chen TJ, et al. (1995) Outcome of very low birth weight infants in industrialized countries: 1947-1987. Am J Epidemiol 141: 1188-1193.
5. Chase HC (1972) A study of infant mortality from linked records: comparison of neonatal mortality from two cohort studies. Rockville, Md: U.S. Department of Health, Education and Welfare. National Center for Health Statistics. Report No. (HSM) 72-1056.
6. Escobar GJ, Littenberg B, Petitti DB (1991) Outcome among surviving very low birthweight infants: a meta-analysis. Arch Dis Child 66: 204-211.
7. Lorenz JM, Wooliever DE, Jetton JR, Paneth N (1998) A quantitative review of mortality and developmental disability in extremely premature newborns. Arch Pediatr Adolesc Med 152: 425-435.
8. Lorenz JM (2001) The outcome of extreme prematurity. Semin Perinatol 25: 348-359.
9. Roberts G, Anderson PJ, Doyle LW, Victorian Infant Collaborative Study Group (2009) Neurosensory disabilities at school age in geographic cohorts of extremely low birth weight children born between the 1970s and the 1990s. J Pediatr 154: 829-834.
10. Saigal S, Doyle LW (2008) An overview of mortality and sequelae of preterm birth from infancy to adulthood. Lancet 371: 261-269.
11. Taylor HG, Klein N, Minich NM, Hack M (2000) Middle-school-age outcomes in children with very low birthweight. Child Dev 71: 1495-1511.
12. Singer RB, Strauss D, Shavelle R (1998) Comparative mortality in cerebral palsy patients in California, 1980-1996. J Insur Med 30: 240-246.
13. Strauss D, Eyman RK (1996) Mortality of people with mental retardation in California with and without Down syndrome, 1986-1991. Am J Ment Retard 100: 643-653.
14. Honeycutt A, Dunlap L, Chen H, Al Homsi G (2000) The cost of developmental disabilities. Research Triangle Park, NC: Research Triangle Institute.
15. Honeycutt AA, Grosse SD, Dunlap LJ, Schedel DE, Chen H, et al. (2003) Economic costs associated with mental retardation, cerebral palsy, hearing loss, and vision impairment. In: Altman B, Barnartt S, Hedershot G, Larson S, editors. Using survey data to study disability: results from the National Health Interview Survey on disability. London: Elsevier. pp. 207-228.
16. Carroll AE, Downs SM (2009) Improving decision analyses: parent preferences (utility values) for pediatric health outcomes. J Pediatr 155: 21-25, 25.
17. World Health Organization. CHOosing Interventions that are Cost-Effective (WHO-CHOICE). Health State Valuations. [Internet]. Available at: http://who.int/choice/demography/health_valuations/en/index.html. Accessed on 19 October 2010.

**Table S1.** Parameter ranges examined in sensitivity analyses.

| Parameter | Lower bound | Upper bound |
|---|---|---|
| **Neonatal mortality probabilities** | | |
| With NICU, 24-26 weeks | 0.23 | 0.75 |
| With NICU, 27-29 weeks | 0.08 | 0.51 |
| With NICU, 30-33 weeks | 0.03 | 0.14 |
| Without NICU | BC x 0.85 | BC x 1.16* |
| **Probabilities of long-term morbidity** | | |
| Minor disability, with NICU | BC x 0.84 | BC x 1.43† |
| Minor disability, without NICU | BC x 0.43 | BC x 1.57† |
| Major disability, with NICU | BC x 0.84 | BC x 1.43 |
| Major disability, without NICU | BC x 0.43 | BC x 1.57 |
| **Relative risks of mortality** | | |
| Minor disability | § | § |
| Major disability | BC x 0.75 | BC x 1.25 |
| **Health-state valuations** | | |
| Minor disability | 0.53 | 1.0 |
| Major disability | 0.19 | 1.0 |
| **Initial hospitalization** | | |
| Days in hospital, survivors | BC x 0.75 | BC x 1.25 |
| Days in hospital, deaths | BC x 0.75 | BC x 1.25 |
| Proportion ventilated days | BC x 0.75 | BC x 1.25 |
| **Nosocomial infection** | | |
| Probability of infection | BC x 0.75 | BC x 1.25* |
| Relative increase in costs | BC x 0.5 | BC x 2 |
| **Rehospitalization days** | BC x 0.75 | BC x 1.25 |
| **Surfactant doses** | BC x 0.75 | BC x 1.25 |
| **Probabilities of surgery** | | |
| Retinopathy of prematurity | BC x 0.75 | BC x 1.25 |
| VP shunt | BC x 0.75 | BC x 1.25 |
| PDA ligation | BC x 0.75 | BC x 1.25 |
| Necrotizing enterocolitis | BC x 0.75 | BC x 1.25 |
| **Unit costs** | | |
| Ventilated bed-day | BC x 0.5 | BC x 2 |
| Non-ventilated bed-day | BC x 0.5 | BC x 2 |
| Surfactant | BC x 0.5 | BC x 2 |
| Retinopathy of prematurity | BC x 0.5 | BC x 2 |
| VP shunt | BC x 0.5 | BC x 2 |
| PDA ligation | BC x 0.5 | BC x 2 |
| Necrotizing enterocolitis | BC x 0.5 | BC x 2 |
| Long-term costs, minor disability | BC x 0.5 | BC x 2 |
| Long-term costs, major disability | BC x 0.5 | BC x 2 |

* Upper bound truncated at 0.99.

† Where probabilities of major and minor disability summed to greater than 1, probability of minor disability was truncated to constrain the sum to 1.

§ In the base-case analysis, we assumed that minor disability produced no excess mortality. In sensitivity analyses, the upper bound reflects an alternative assumption that the excess risk (RR – 1) for minor disability was half the base-case (RR – 1) for major disability. The lower bound was equal to the base case assumption that RR = 1.0.

Abbreviations: NICU – neonatal intensive care unit; GA – gestational age; BC – base-case value; VP – ventriculo-peritoneal; PDA – patent ductus arteriosus; RR – relative risk.

**Table S2.** Results from univariate sensitivity analyses.

| | Incremental cost-effectiveness of NICU vs. no NICU* | | | | | |
|---|---|---|---|---|---|---|
| | 24-26 weeks GA | | 27-29 weeks GA | | 30-33 weeks GA | |
| **Parameter** | **At lower bound** | **At upper bound** | **At lower bound** | **At upper bound** | **At lower bound** | **At upper bound** |
| **Neonatal mortality probabilities** | | | | | | |
| With NICU | 1,034 | 1,762 | 613 | 753 | 245 | 238 |
| Without NICU | 1,561 | 1,158 | 656 | 644 | 200 | 276 |
| **Probabilities of long-term morbidity** | | | | | | |
| Minor disability, with NICU | 1,202 | 1,313 | 627 | 710 | 230 | 269 |
| Minor disability, without NICU | 1,245 | 1,222 | 664 | 636 | 272 | 214 |
| Major disability, with NICU | 1,156 | 1,333 | 600 | 790 | 185 | 388 |
| Major disability, without NICU | 1,259 | 1,210 | 714 | 592 | 471 | 51 |
| **Relative risks of mortality** | | | | | | |
| Minor disability† | 1,232 | 1,311 | 648 | 672 | 241 | 241 |
| Major disability | 1,246 | 1,220 | 661 | 638 | 237 | 245 |
| **Health-state valuations** | | | | | | |
| Minor disability | 1,485 | 1,190 | 742 | 632 | 240 | 241 |
| Major disability | 1,349 | 1,159 | 685 | 624 | 238 | 244 |
| **Initial hospitalization** | | | | | | |
| Days in hospital, survivors | 1,117 | 1,347 | 571 | 726 | 182 | 301 |
| Days in hospital, deaths | 1,214 | 1,250 | 641 | 656 | 238 | 244 |
| Proportion ventilated days | 1,211 | 1,252 | 639 | 657 | 237 | 245 |
| **Nosocomial infection** | | | | | | |
| Probability of infection | 1,213 | 1,237 | 643 | 654 | 238 | 244 |
| Relative increase in costs | 1,197 | 1,309 | 638 | 670 | 236 | 253 |
| **Rehospitalization days** | 1,225 | 1,239 | 645 | 652 | 239 | 243 |
| **Surfactant doses** | 1,223 | 1,240 | 647 | 650 | 239 | 243 |
| **Probabilities of surgery** | | | | | | |
| Retinopathy of prematurity | 1,228 | 1,236 | 648 | 649 | 241 | 241 |
| VP shunt | 1,231 | 1,233 | 648 | 649 | 241 | 241 |
| PDA ligation | 1,174 | 1,290 | 636 | 661 | 237 | 245 |
| Necrotizing enterocolitis | 1,228 | 1,235 | 647 | 650 | 240 | 242 |
| **Unit costs** | | | | | | |
| Ventilated bed-day | 1,072 | 1,552 | 576 | 793 | 210 | 304 |
| Non-ventilated bed-day | 1,112 | 1,471 | 544 | 858 | 144 | 435 |
| Surfactant | 1,215 | 1,266 | 645 | 655 | 237 | 250 |
| Retinopathy of prematurity | 1,224 | 1,248 | 648 | 649 | 241 | 241 |
| VP shunt | 1,230 | 1,236 | 648 | 650 | 241 | 241 |
| PDA ligation | 1,116 | 1,463 | 624 | 698 | 233 | 258 |
| Necrotizing enterocolitis | 1,225 | 1,246 | 646 | 654 | 239 | 245 |
| Long-term costs, minor disability | 1,174 | 1,348 | 605 | 735 | 242 | 239 |
| Long-term costs, major disability | 1,104 | 1,488 | 576 | 793 | 262 | 199 |

* 2005 US dollars per disability-adjusted life year

† Lower bound reflects base-case assumption that minor disability produces no excess mortality.

Abbreviations: NICU – neonatal intensive care unit; GA – gestational age; VP – ventriculo-peritoneal; PDA – patent ductus arteriosus.

**Figure S1.** Results from univariate sensitivity analyses, by gestational age group

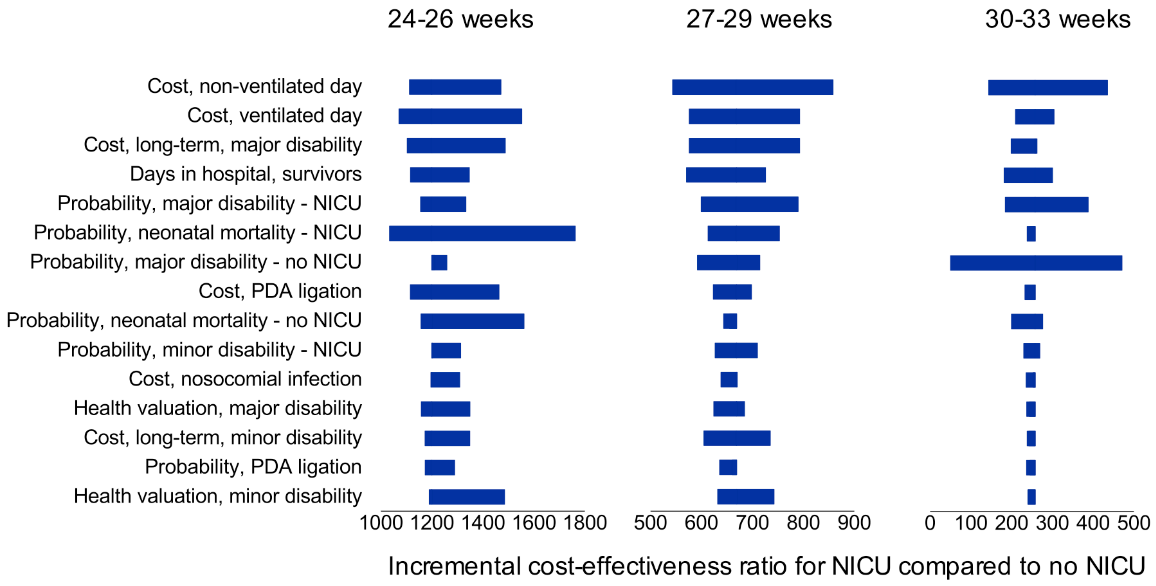


**Figure S2.** Cost-effectiveness acceptability curves for neonatal intensive care compared to no neonatal intensive care, by gestational age group.

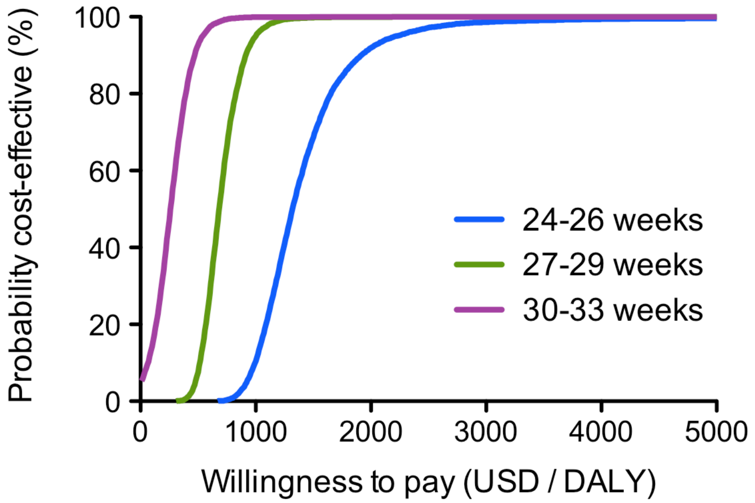

Supplement: Text S1 — Technical appendix. The supporting tables and figures are available as individual files (Figures S1 and S2 and Tables S1 and S2), but are also included here for ease of access. (0.46 MB PDF) [file pmed.1000379.s005.pdf]
